# Clamp conformational flexibility and dynamics in archaeal and eukaryotic RNA polymerases revealed by cryo-EM

**DOI:** 10.1016/j.jbc.2026.113389

**Published:** 2026-08-04

**Authors:** George N.R. Fordjour, Leon Palao, Kenji Murakami, Jean-Paul Armache, Katsuhiko S. Murakami

**Affiliations:** 1 Department of Biochemistry and Molecular Biology, Penn State University, University Park, Pennsylvania, USA; 2 Huck Institutes of the Life Sciences, Center for Structural Biology, Penn State University, University Park, Pennsylvania, USA; 3 Huck Institutes of the Life Sciences, Center for RNA Molecular Biology, Penn State University, University Park, Pennsylvania, USA; 4 Huck Institutes of the Life Sciences, Center for Eukaryotic Gene Regulation, Penn State University, University Park, Pennsylvania, USA; 5 Molecular Machines Mechanism and Structure Predoctoral Training Program, Penn State University, University Park, Pennsylvania, USA; 6 Department of Biochemistry and Biophysics, Perelman School of Medicine, University of Pennsylvania, Philadelphia, Pennsylvania, USA

**Keywords:** cellular RNA polymerase, cryo-electron microscopy, conformational dynamics, promoter complex, compositional heterogeneity, *in silico* purification, structural biology

## Abstract

All cellular RNA polymerases (RNAPs) across Bacteria, Archaea, and Eukarya share a conserved catalytic core, yet bacterial and archaeal-eukaryotic RNAPs diverged after separation from the last universal common ancestor. This evolutionary split produced distinct subunit compositions and fundamentally different requirements for external factors during transcription initiation. Bacterial RNAP relies on a σ factor, whereas archaeal-eukaryotic RNAPs require a more extensive set of general transcription factors (GTFs) to bind promoter DNA, unwind the duplex, and position the template strand within the active site cleft. Notably, despite the close structural similarity between archaeal and eukaryotic RNAPs, the requirement for GTFs became further specialized after the emergence of Eukarya. This divergence raises the question of whether differences in intrinsic conformational flexibility and dynamics of these RNAPs contribute to distinct promoter-loading pathways. In this study, we addressed this question using cryo-electron microscopy (cryo-EM) to examine archaeal RNAPs from Euryarchaeota and Crenarchaeota alongside yeast RNAP II. Archaeal RNAP displays a highly dynamic DNA binding clamp domain that samples a broad spectrum of open and closed states, whereas RNAP II predominantly adopts a closed clamp state. Both archaeal and eukaryotic RNAPs can be found in stalk-bound and stalk-less forms. Comparative structural analyses further reveal a unique conformational transition in crenarchaeal RNAP associated with clamp opening. Together, these findings define the intrinsic clamp-conformational landscapes across the archaeal-eukaryotic lineage and suggest that evolutionary tuning of clamp flexibility and dynamics contributes to distinct GTF-dependent promoter-loading mechanisms.

Cellular RNA polymerases (RNAPs) are responsible for transcribing DNA into RNA across all three domains of life. Bacterial RNAP contains five subunits (α_2_ββ’ω) responsible for catalytic core enzyme formation and all subunits are conserved throughout evolution (1, 2). Structurally, cellular RNAPs adopt a conserved crab-claw architecture, with two pincers that accommodate the template DNA and a bottom of cleft that houses the active site for RNA synthesis (3). The clamp domain, one of the key structural elements of cellular RNAP, undergoes conformational changes to regulate template DNA access to the active site, thus plays a critical role in all stages of the transcription cycle (4, 5).

The clamp transitions from the closed to open states, which widens the DNA binding cleft, facilitating promoter DNA loading, and subsequent closing of the clamp stabilizes the open complex. The clamp also serves as one of the platforms for assembly of RNAP binding factors such as σ (6) and N-utilization substance G (7) in bacterial RNAP, and some general transcription factors (GTFs) (8, 9, 10) and suppressor of Ty (Spt)4/5 elongation factor (also known as DRB sensitivity-inducing factor) in archaeal-eukaryotic RNAPs (5, 11, 12). The functional importance of the clamp is further underscored by small-molecule bacterial RNAP inhibitors such as myxopyronin and lipiarmycin, which bind to a hinge region of the clamp and restrict its rotation to block transcription, thus limiting bacterial cell growth (13, 14).

RNAP II in eukaryotes (eRNAP II), which is responsible for messenger RNA (mRNA) and regulatory RNA transcription, is the most functionally and structurally closely related to archaeal RNAP (aRNAP) (15). One of the distinguishing features of these RNAPs as compared to bacterial RNAP is having a stalk domain consisting of heterodimeric subunits (Rpo4/Rpo7 in aRNAP; Rpb4/Rpb7 in eRNAP II), which interact with the Rpo6/Rpb6 subunit and a hinge region of the clamp (Fig. S1). Early X-ray crystallographic studies of eRNAP II from *Saccharomyces cerevisiae* (baker's yeast) suggested that the absence of the stalk leads to an open clamp conformation (16, 17), implying that the stalk controls clamp dynamics. However, subsequent X-ray studies of aRNAP from *Saccharolobus solfataricus* (Crenarchaeota) (15) and *Thermococcus kodakarensis* (*Tko*, Euryarchaeota) (18), as well as cryo-EM study of *Pyrococcus furiosus* (*Pfu*, Euryarchaeota) (19), demonstrated that aRNAP with an intact stalk can adopt both open and closed clamp states.

Genome organization and regulatory complexity differ profoundly between eukaryotes and archaea. Eukaryotic genomes are generally larger, characterized by lower gene density, and sequestered within complex chromatin (20). This architecture requires sophisticated regulatory layers, including ATP-dependent chromatin remodeling complexes and diverse epigenetic histone tails and/or DNA base modifications, which are largely absent or significantly simplified in archaeal systems (21). Despite these divergent genome organizational strategies, the fundamental mechanism of basal transcription remains strikingly conserved. Archaeal RNAP exhibits remarkable structural similarity to eukaryotic RNAP II; both systems utilize GTFs for promoter recognition, pre-initiation complex (PIC) assembly, DNA melting, and transcription initiation. However, the required number of GTFs varies. While eukaryotic systems require an extensive suite of GTFs (TFIIA, B, D, E, F, H including TATA-binding protein (TBP)-associated factors TAFs in TFIID), archaeal transcription initiation necessitates only a minimal set: TBP, Transcription Factor B (TFB), and Transcription Factor E (TFE) (2, 8, 22).

Previous biochemical and structural studies of aRNAP and eRNAP II have demonstrated their highly conserved subunit composition and 3D structure (Fig. S1) (1, 15, 23, 24). However, these studies have not resolved why eRNAP II requires so many GTFs for promoter-specific transcription. Since the clamp domain of RNAP must adapt to both closed and open states during transcription initiation, a likely explanation may stem from the intrinsic flexibility (range of widening the clamp) and dynamics (equilibrium between closed and open states) of aRNAP and eRNAP II, a property that was not being addressed systematically. While structural studies of eRNAP II have been extensively pursued using cryo-EM, the apo-form has not yet been characterized (25). Therefore, we aim to fill that gap, providing new insights into the flexibility in the apo-form eRNAP II and enhancing our understanding of its function. Macromolecular structure determination by cryo-EM can provide information that other structural methods cannot readily access, such as compositional heterogeneity and structural flexibility and dynamics (7, 19, 26). In this study, we addressed this question by using cryo-EM single-particle reconstruction and 3D classification in the apo-form of aRNAPs from *Pfu* and *Sulfolobus acidocaldarius* (*Sac*, Crenarchaeota), as well as eRNAP II from *S. cerevisiae*. *Pfu* and *Sac* aRNAPs were selected because these cells can be cultured on a large scale for their aRNAP preparations and well-studied species that represent distinct evolutionary lineages, enabling comparative analysis of feature conservation across diverse archaeal taxa.

## Results

### Cryo-EM structure determination of the archaeal RNAPs and eukaryotic RNAP II

**Figure 1:**
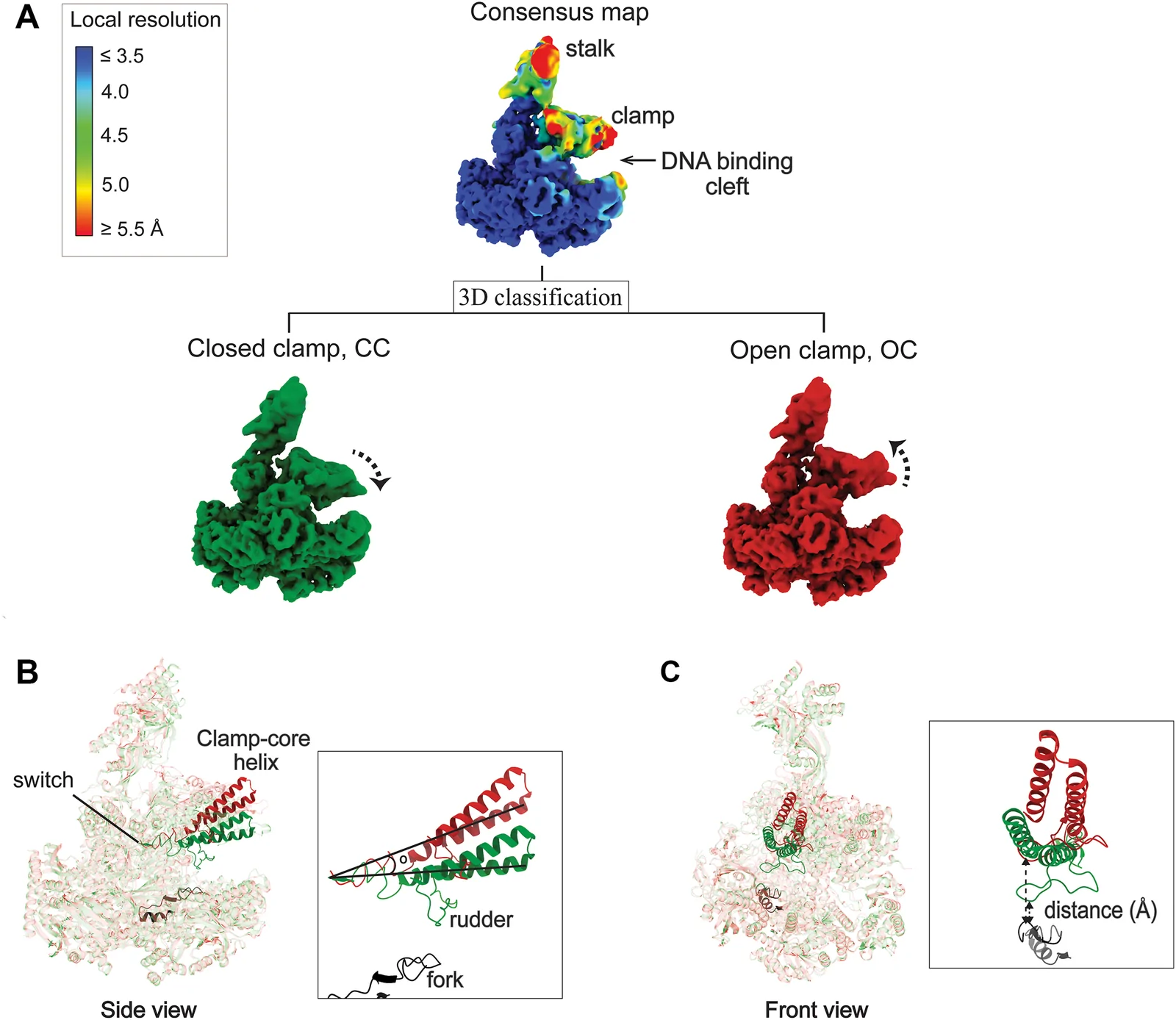
**Experimental scheme to evaluate flexibility and dynamics of the clamp domain of cellular RNAP by cryo-EM data analysis.***A*, (*top*) local-resolution map of a representative RNAP (*Pfu* aRNAP) prior to 3D classification, highlighting conformational heterogeneity evident as lower local resolution in the clamp and stalk regions relative to the main body. (*bottom*) After 3D classification with a focused mask around the clamp reconstituted cryo-EM maps representing either closed (*green*) or open clamp domain (*red*). Superposition of the closed-clamp (*green*) and open-clamp (*red*) models in side view (*B*) and front view (*C*). Residues of clamp-core helix and rudder through to the switch region, and the fork of the RNAP are highlighted in solid colors whereas other RNAP regions are made transparent. Clamp opening angle (°) was measured at the distal tip of the clamp-core helices to the hinge (“switch”) region. Cleft width was measured as the minimum distance between the rudder and fork loop 1 (Å).

To quantify flexibility and dynamics of RNAP and compare these features associated with three RNAPs, we maintained consistent experimental procedures, including cryo-EM specimen preparation, data collection and processing throughout this study (Figs. S2–S4). Cellular RNAP exhibits preferred particle orientation due to air-water interface, limiting certain views of particles and hampering not only resolution but also the estimation of heterogeneity and dynamics (9, 27). This issue was mitigated by adding CHAPSO to the samples before vitrification (28). Additionally, to preserve native state of these RNAPs, we avoided the use of chemical crosslinkers during specimen preparation. Cryo-EM data for all three RNAPs were collected using a Talos Arctica G2 200 kV microscope equipped with a Falcon 4 direct electron detector and motion-corrected in CryoSPARC v.4.1.2 (29). Particles were automatically picked using Topaz in CryoSPARC (30). Particles were refined into 2D classification followed by reference-free *ab initio* 3D reconstruction. Subsequent 3D refinements (heterogenous and non-uniform refinements) enabled us to enrich particles belonging not only to each targeted macromolecule but also to distinguished subunit composition and conformation, a process we term “*in silico* purification” (31). This approach was particularly valuable for the present analysis, as attempts to separate stalk-bound and stalk-less *Sac* aRNAPs by size-exclusion chromatography were unsuccessful due to their similar molecular weights (Fig. S5). In all three RNAPs, the cryo-EM density around their stalks (Rpo4/7 in aRNAP and Rpb4/7 in yeast eRNAP II) were less well-defined compared with their main bodies (Fig. 1*A*), suggesting their sub-stoichiometric occupancy. Heterogenous refinement using 3D maps of RNAP with or without stalk revealed two subsets of particles in which stalk was either fully present (stalk-bound form RNAP) and completely absent (stalk-less RNAP) (Figs. S2–S4). The stalk-bound forms of *Pfu* aRNAP, *Sac* aRNAP, and yeast eRNAP II were reconstructed at nominal resolutions of 3.9 Å, 3.4 Å, and 3.3 Å, respectively. Cryo-EM maps of stalk-less RNAPs yielded lower resolutions due to the smaller number of particles, with nominal resolutions of 4.6 Å (*Pfu* aRNAP), 8.9 Å (*Sac* aRNAP), and 6.8 Å (yeast eRNAP II) (Figs. S2–S4 and Tables S1–S3).

### The DNA-binding clamp domain flexibility and dynamics of archaeal RNAPs and eukaryotic RNAP II

In the cryo-EM density maps of these RNAPs, local resolution around the clamp was substantially lower compared with other areas, suggesting conformational heterogeneity of the clamp (Fig. 1*A* top). We thus performed 3D Variability Analysis (3DVA) in CryoSPARC (26), which revealed continuous conformational change of the clamp. This analysis showed a swinging motion of the clamp between closed and open states, thereby changing the DNA-binding cleft width. To further characterize the flexibility and dynamics, we performed 3D classification with a focused mask (32) covering the clamp (Figs. S2–S4). The resulting maps of both the closed and open states allowed us to refine the 3D structure with high-quality cryo-EM densities (Fig. 1*A* bottom). This analysis reveals not only the structures of two distinct clamp conformation (heterogeneity) but also quantifies the distribution of RNAP between the two conformations (dynamics). We examined only the stalk-bound form of RNAPs since the stalk-less form of RNAPs does not have sufficient numbers of particles for the 3D classification followed by getting high-quality cryo-EM densities for the 3D structure refinements.

To quantify the clamp flexibility of each RNAP, we measure two key parameters from the final 3D refined structures: the angle of clamp rotation between the closed and open states (Fig. 1*B*) and the distance between the rudder and the fork in each clamp conformation (Fig. 1*C*), which together define the boundaries of the DNA-binding cleft. Clamp rotation was measured by identifying structurally conserved residues in all three RNAPs at the distal tip of the coiled-coil helix and the switch region. Similarly, the distance of the DNA-binding cleft was determined using conserved residues located on the rudder and fork (Fig. S6). For this measurement, residues were selected based on their minimal separation between the rudder and fork, providing a consistent metric that defines the narrowest point of the cleft and directly reflects the spatial constraint for DNA binding. Dynamics of the clamp were addressed by counting the number of particles belonging to each state of clamp (Figs. S2–S4 and Tables S1–S3).

**Figure 2:**
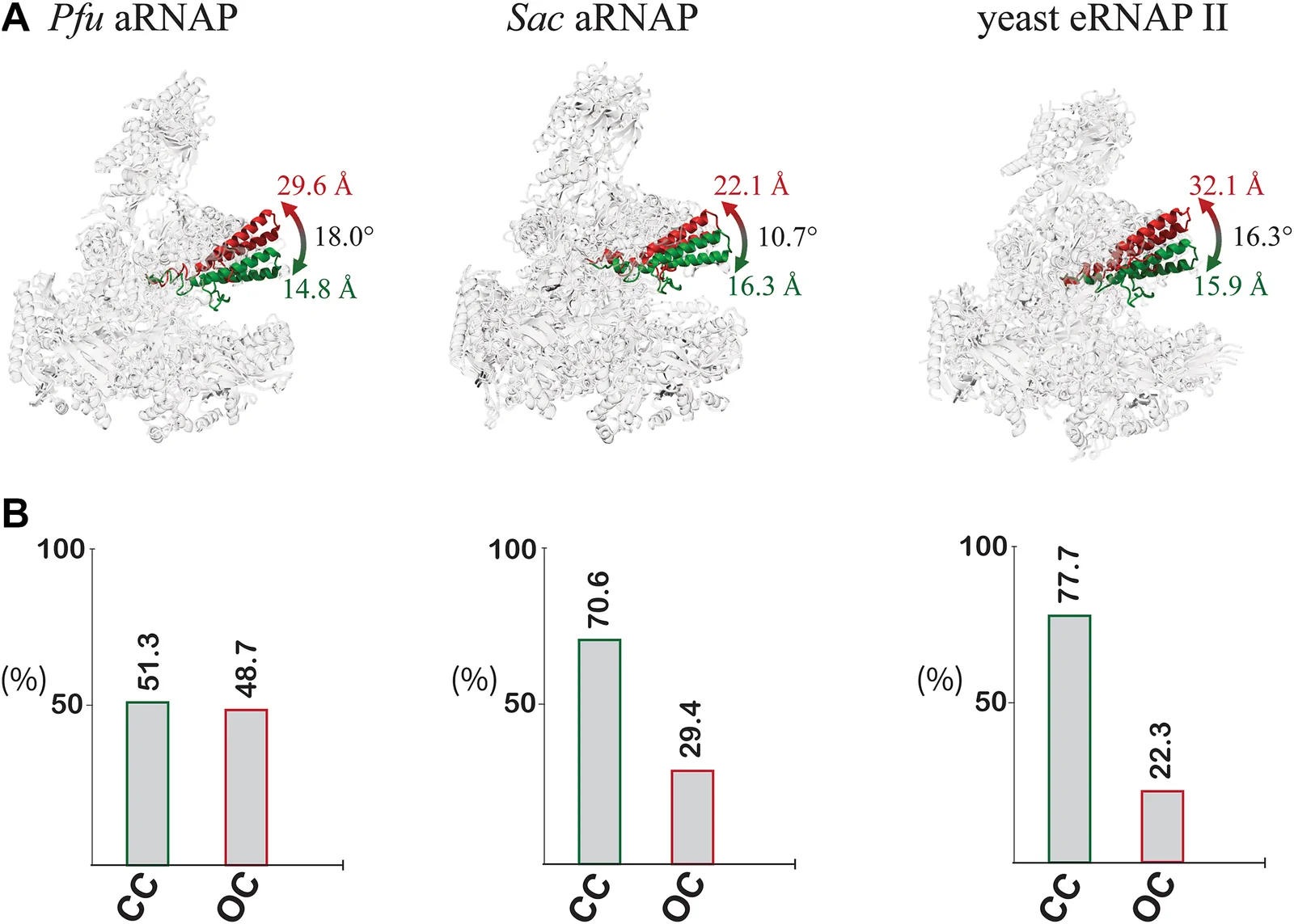
**Conformational dynamics of the clamp of archaeal RNAPs and eukaryotic RNAP II.***A*, superimposed structures of the closed and open clamp conformations in stalk-bound RNAPs from *Pfu* (*left*), *Sac* (*middle*), and yeast (*right*). The clamp domains of these RNAPs are highlighted in *green* (closed clamp, CC) and *red* (open clamp, OC). Angle between the closed and open clamp states, distances of the DNA-binding cleft in either the closed or open clamp states are indicated. Residues used for all angle and distance measurements are provided in Fig. S6 (*B*) Distribution of RNAP particles adopting the closed (*green*) and open (*red*) clamp states (*left*, *Pfu*; *middle*, *Sac*; *right*, yeast). RNAPs are shown in side views.

Among three RNAPs, the clamp of *Pfu* aRNAP is the most dynamic and highly flexible as it rotates 18.0° from the closed to the open states, expanding the DNA-binding cleft from 14.8 Å (closed state) to 29.6 Å (open state) (Fig. 2*A*). Nearly equal numbers of *Pfu* aRNAP molecules belong to these states (51% in the closed and 49% in the open states) (Fig. 2*B*). *Sac* aRNAP is less flexible as it expands the DNA binding cleft to only 22.1 Å in the open state with 10.7° rotation between these two states (Fig. 2*A*) and is less dynamic as two-third of *Sac* aRNAP molecules adopted the closed state (Fig. 2*B*). The yeast eRNAP II exhibited behavior intermediate between *Pfu* and *Sac* aRNAPs. Its clamp is flexible as the *Pfu* aRNAP (the DNA-binding cleft of eRNAP II widens 32.1 Å in the open state) (Fig. 2*A*), but less dynamic as only 22% of yeast eRNAP II molecules belonging to the open state (Fig. 2*B*).

Several cryo-EM structures of archaeal RNAP in both closed- and open-clamp states have recently been determined (9, 19, 33). We also measured their DNA-binding cleft widths and summarized alongside the structures we determined, enabling direct comparison across archaeal RNAPs (Table S4). The closed-clamp state of the *Pfu* aRNAP and *Sac* aRNAP determined from this study resembles the conformation of the *Tko* aRNAP (apo-form) and the *Pfu* aRNAP (contracted form or elongation complex), whereas the open-clamp state resembles to the *Tko* aRNAP bound to TFE, the *Pfu* aRNAP (expanded form) or the *Sac* aRNAP bound to transcription inhibitor TFS4.

**Figure 3:**
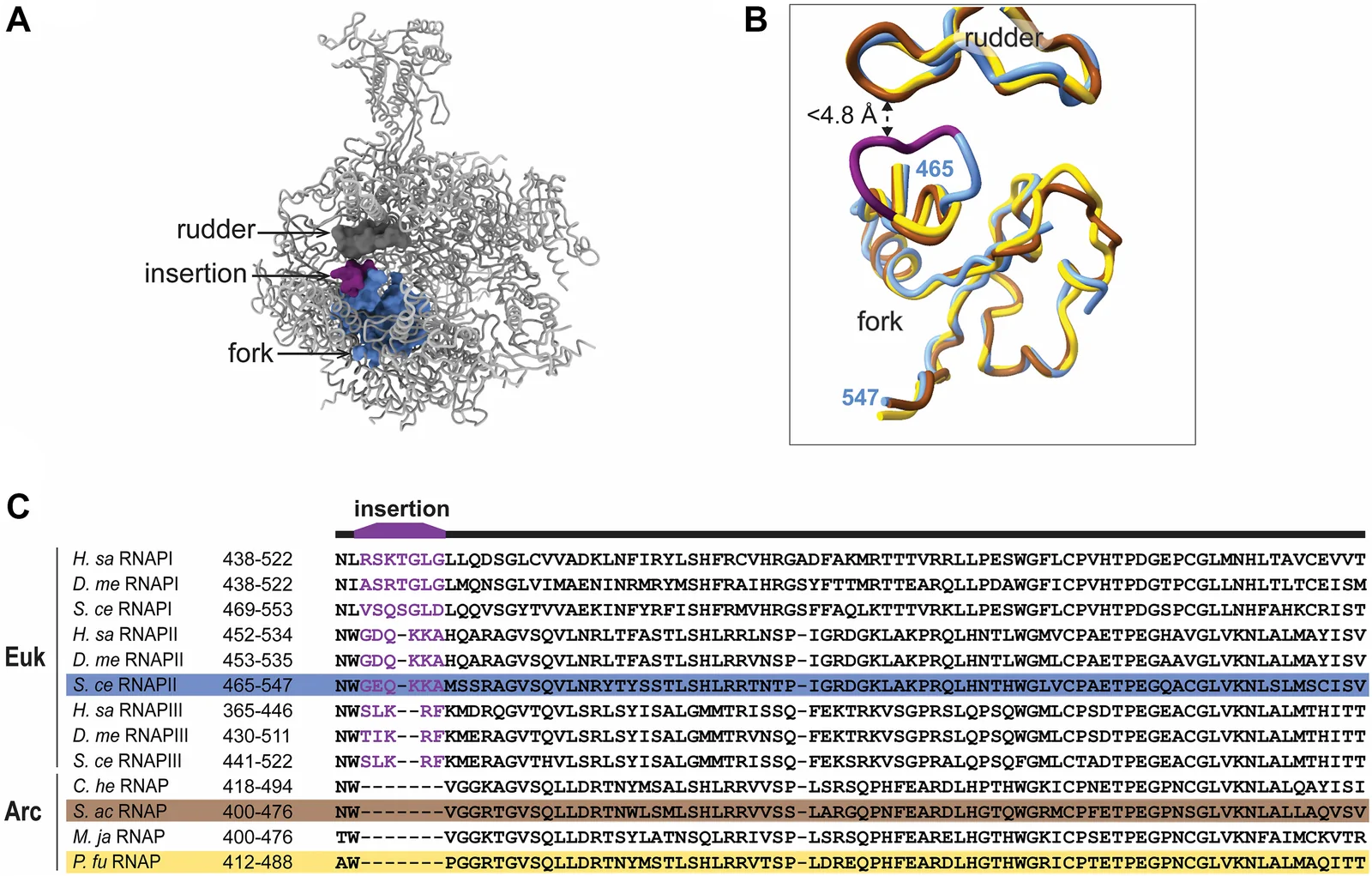
**Structural and sequence analysis of the fork loop 1 of aRNAP and eRNAP II.***A*, overall structure of yeast eRNAP II with the Rpb2 fork region shown in *blue*, the fork insertion (*violet*), and the rudder (*gray*). RNAP is shown in front view. *B*, structural superposition of the fork region and rudder from yeast Rpb2 (colored as in *A*), *Pfu* Rpo2 (*yellow*), and *Sac* Rpo2 (*orange*). The 6-amino acid insertion in *S. cerevisiae* eRNAP II projects into the cleft and reduces the minimal gap distances between the fork region and the rudder to <4.8 Å. *C*, sequence alignment of the fork loop 1 region from representative eukaryotic (Euk) and archaeal (Arc) RNAPs (H. sa, *Homo sapience*; D. me, *Drosophila megalogaster*; S. ce, *Saccharomyces cerevisiae*; C. he, *Candidatus heimdallarchaeota* [Asgardarchaeota]; S. ac, *Sulfolobus acidocaldarius*; M. ja, *Methanocaldococcus jannaschii*; P. fu, *Pyrococcus furiosus*). Residues corresponding to the insertion are highlighted in violet. Background colors for the yeast *Pfu* and *Sac* sequences match *panel B*.

From comprehensive structural analysis of three RNAPs, we identified a unique structural feature only found in yeast eRNAP II: the Rpb2 fork loop 1 contains a six-amino-acid insertion that projects into the upstream DNA-binding cleft and constricts the cleft to a width < 4.8 Å from a pincer opposite side from the clamp domain (Fig. 3, *A* and *B*). This insertion is conserved in eukaryotic RNAP II from yeast to human but absent in archaeal RNAPs, including *Candidatus heimdallarchaeota*, a class of archaea within the phylum Asgardarchaeota, which is the closest known archaeal relative to eukaryotes (Fig. 3*C*), suggesting its role as an additional steric barrier that must be displaced during DNA loading and potentially requiring a more pronounced widening of the upstream cleft compared to archaeal RNAP. The Rpb2 fork loop 1 also plays a role in stabilizing the upstream edge of the transcription bubble (34).

### Structural changes of RNAP associated with the conformational change of clamp

**Figure 4:**
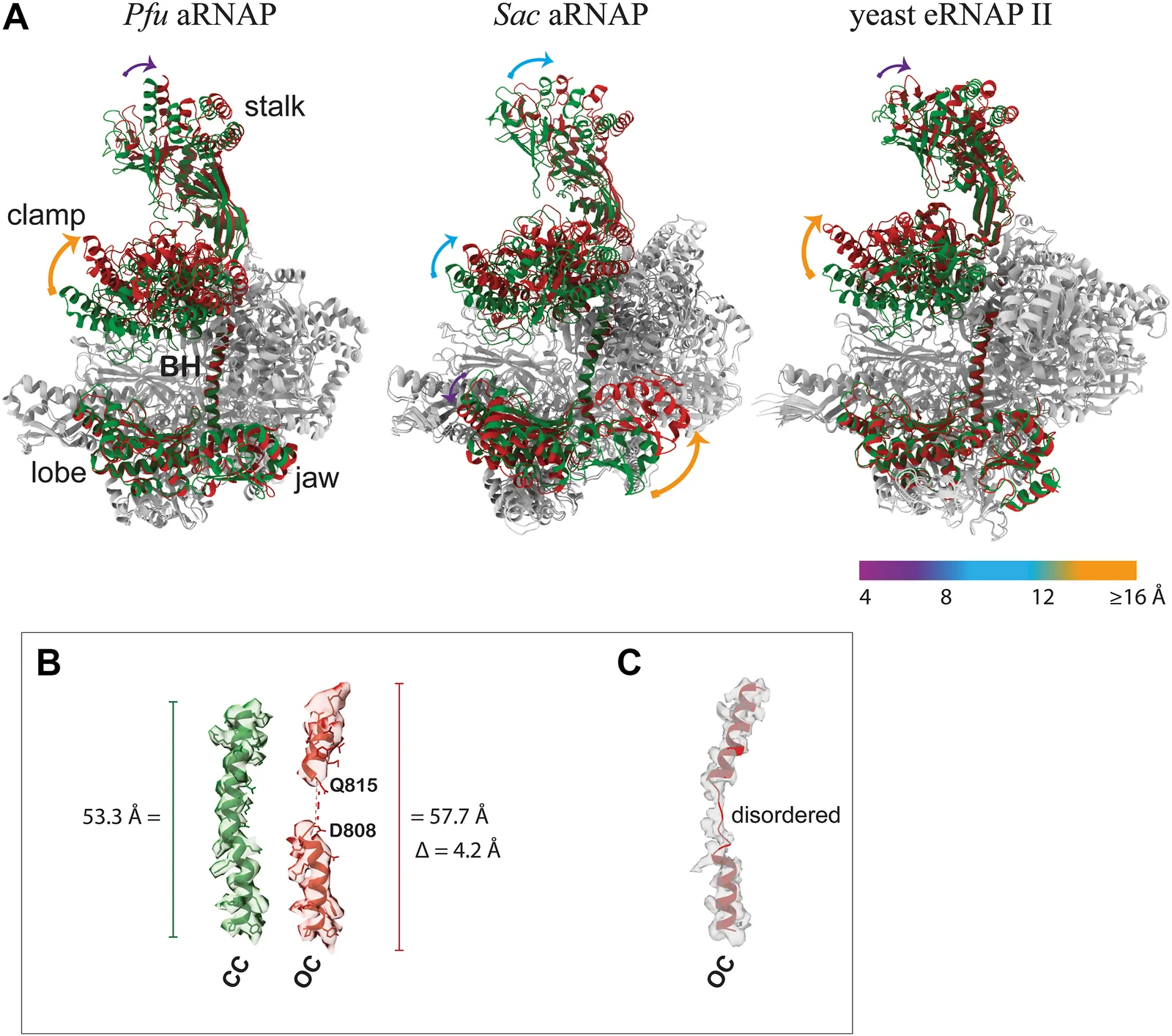
**Structural changes of RNAP associated with the clamp opening.***A*, superposition of RNAP structures in the closed-clamp (*green*) and open-clamp (*red*) states for *Pfu* aRNAP (*left*), *Sac* aRNAP (*middle*), and yeast eRNAP II (*right*). The RNAP core is shown in *gray*; regions that undergo large rearrangements during clamp opening are colored by the magnitude of displacement between states (Å; color scale). Quantitative measurements of the movements of individual RNAP modules are shown in Fig. S8. *B*, Bridge helix (BH) conformations in the closed (*green*) and open (*red*) states of *Sac* aRNAP. In the open-clamp state, the central segment of the BH is disrupted, producing a discontinuity near residues D808-Q815 and an overall elongation of the BH axis (Δ = 4.2 Å). *C*, local refinement of the BH region in *Sac* aRNAP showing loss of a continuous α-helix and formation of a looped/disordered segment in the open-clamp state.

**Figure 5:**
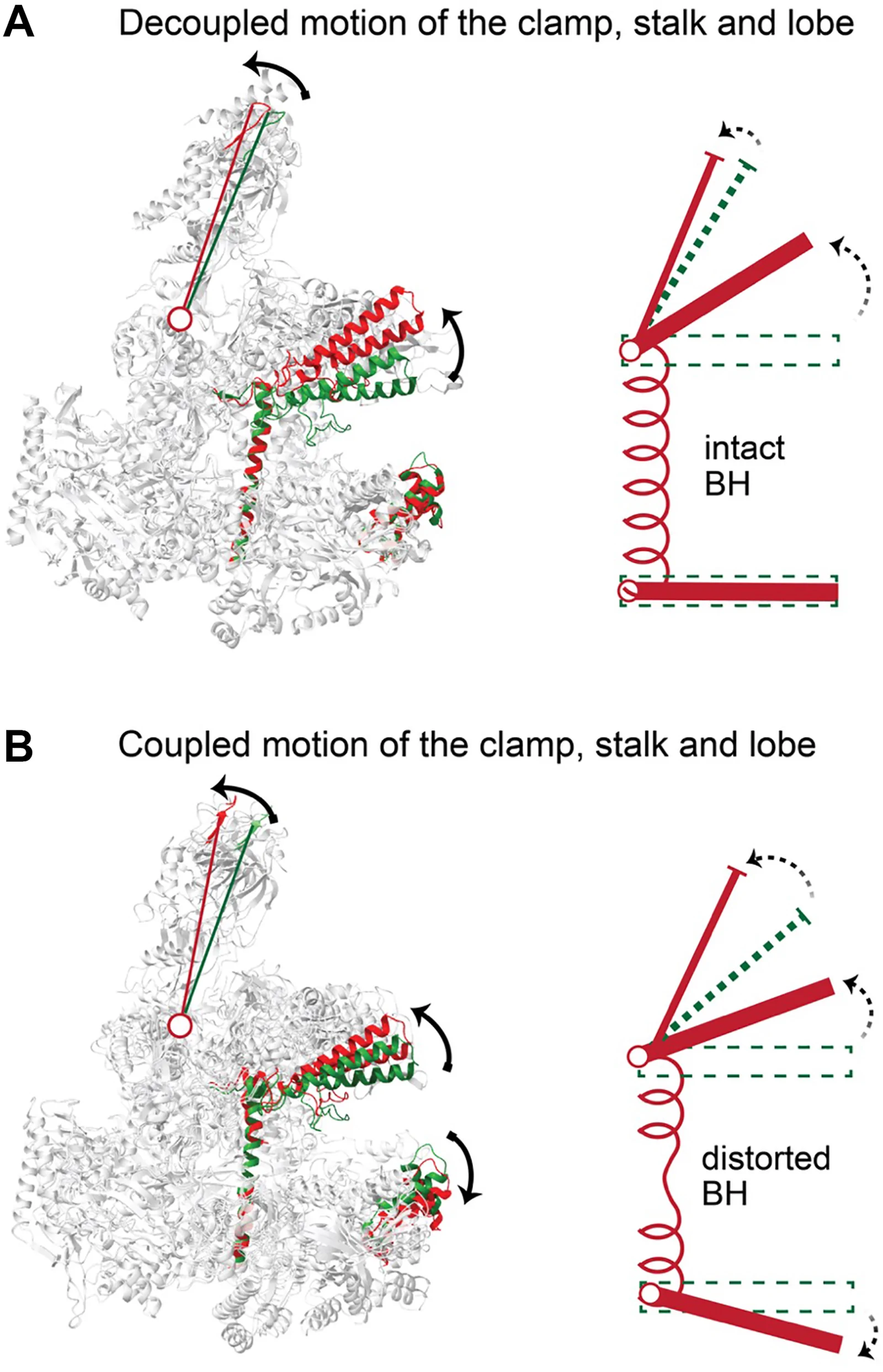
**Schematic depiction of RNAP conformational changes associated with clamp opening.***A*, in *Pfu* aRNAP and yeast eRNAP II, opening of the clamp is primarily achieved by swinging the clamp outward, with only modest accompanying shifts of the stalk and lobe. The bridge helix (BH) remains largely intact and is depicted as a spring connecting the two pincers. *B*, in *Sac* aRNAP, opening of the DNA-binding cleft is accompanied by coordinated motions of multiple structural elements, including the clamp, stalk, lobe, and jaw. In this case, the BH is distorted, consistent with coupled rearrangements of the RNAP pincers. Closed- and open-clamp states are shown in *green* and *red*, respectively.

Next, we investigated overall structural changes of RNAPs associated with their clamp conformational change (Figs. 4, 5 and S6–S8). In the case of *Pfu* aRNAP and yeast eRNAP II, their clamps simply swing to widen the DNA-binding cleft with subtle shifts in the position of their stalks (Fig. 5*A*). These movements did not influence the conformation of the bridge helix (Fig. 4*A*), which positions itself in the middle of RNAP, connecting the two pincers of the DNA-binding cleft, including the clamp and the protrusion/lobe/jaw domains. Accordingly, both RNAPs showed modest movement in their stalk/jaw/lobe domains upon clamp opening (Figs. 4*A* and S8).

In sharp contrast, *Sac* aRNAP exhibits four prominent structural rearrangements during the clamp opening. The DNA-binding cleft widens by swinging not only the clamp but also the side of pincer including protrusion/lobe/jaw domains (Figs. 4*A* and S8). As a result, the bridge helix stretches, leading to the loss of its cryo-EM density at the central region (residues Asp808 to Gln815) when the DNA-binding cleft widens (Fig. 4*B*). Local refinement around the bridge helix restored the density, revealing an extended loop, resembling a stretched spring (Fig. 4*C*). The Rpo1C-jaw domain, which connects the two pincers by interacting with the Rpo2-lobe domain in the closed clamp state, moves 17.7 Å upward upon clamp opening, creating a large gap between the jaw and lobe domains (Fig. S8). This wide gap facilitates binding with TFS4, a potent *Sac* aRNAP inhibitor expressed upon viral infection (33). *Sac* aRNAP moves the stalk together with the clamp, Rpo1-C jaw, Rpo5, Rpo6, and Rpo13 subunits, resulting in approximately one-third of its molecular mass moving as a unit during the opening of DNA-binding cleft (Fig. 5*B*).

## Discussion

Since the pioneering biochemical characterization of archaeal RNAP by Wolfram Zillig and Karl Stetter, which revealed its eukaryotic-like subunit composition and reliance on GTFs for promoter complex formation (23), a longstanding question has been why archaeal RNAP requires only a minimal set of GTFs, whereas eukaryotic RNAP II depends on a large and complex initiation machinery. Subsequent X-ray crystallographic studies confirmed the remarkable structural similarity between archaeal RNAP and eukaryotic RNAP II (15, 24), further deepening the paradox between conserved RNAP architecture and divergent requirements for transcription factors. What these earlier studies could not capture, however, were the intrinsic conformational heterogeneity and dynamics of cellular RNAPs. In this work, cryo-EM single-particle analysis combined with extensive 3D classification allowed us to visualize and quantify this information on archaeal and eukaryotic RNAPs, providing new insight into their evolutionary divergence.

Across *Pfu* aRNAP, *Sac* aRNAP, and yeast RNAP II, we observed closed and open clamp conformations associated with DNA-binding cleft accessibility. Bacterial (*Escherichia coli*) RNAP predominantly adopts an open clamp conformation in solution (4, 35). Archaeal RNAPs exhibit a broader dynamic range of clamp conformations, with *Pfu* aRNAP showing a preference for the open state (Fig. 2). In contrast, *Sac* aRNAP favors the closed clamp conformation, resembling eukaryotic RNAP II. This difference may reflect evolutionary relationships, as crenarchaeota such as *Sulfolobus* are more closely related to eukaryotes than euryarchaeota such as *Pyrococcus* (20). Our cryo-EM analysis of archaeal RNAPs and eukaryotic RNAP II reveals that clamp dynamics are conserved across all cellular RNAPs.

Several studies have established that the stalk domain of RNAP is a modular feature that plays an important role in survival under environmental stress. In yeast, the stalk is not a constitutive component of every RNAP II molecule; rather, it is present in only a fraction of RNAP II pool and is specifically required for responses to temperature stress, and starvation (36, 37). Similarly, in the archaeon *T. kodakarensis*, the stalk subunit Rpo4 (also known as RpoF) is dispensable for viability at moderate temperatures (38). In contrast, RNAPs lacking stalk domain remain functional *in vitro*, as archaeal RNAP lacking the stalk is capable of transcription initiation, elongation, and termination (38, 39). Biochemical studies in *S*. *cerevisiae* further demonstrate that RNAP II lacking the stalk retains basal catalytic activity *in vitro*, particularly on non-specific DNA templates, but is compromised in promoter-dependent transcription and overall efficiency (37, 40).

In this study, cryo-EM based *in silico* purification further revealed that aRNAP exists in two subpopulations, with and without the Rpo4/7 stalk domain, as previously observed for yeast and *Drosophila* eRNAP II (31, 41). The stalk interacts with factors involved in transcription initiation including TFE/TFIIE (5, 9) as well as with the transcription termination factor FttA factors (42). Although archaeal and eukaryotic RNAPs are generally assumed to contain a complete stalk, several studies including this work provide direct evidence that a substantial population of stalk-less RNAP exists. This finding raises the possibility that stalk-less form represents a physiologically relevant RNAP state rather than merely a consequence of sample preparation. Future studies of archaeal and eukaryotic transcription should therefore consider the abundance, regulation, and potential functional roles of stalk-less RNAP populations.

Among the archaeal RNAPs examined in this study, *Sac* aRNAP exhibited the most extensive overall structural rearrangement during the clamp opening. Unlike *Pfu* aRNAP and yeast RNAP II, which open the cleft primarily through simple clamp rotation, the *Sac* aRNAP uses coordinated movements of the clamp, stalk, lobe, and Rpo1 jaw domains, accompanied by pronounced distortion of the bridge helix into a looped-out conformation (Figs. 4 and 5). This unique behavior underscores that future study of archaeal RNAPs must integrate comparative analyses across diverse archaeal lineages rather than relying on a single model enzyme. Our cryo-EM findings extend single-molecule studies of RNAP clamp dynamics, which have reported spontaneous fluctuations throughout the transcription cycle (4, 5). While these earlier analyses focused predominantly on clamp motion, our work demonstrates that cleft opening in some RNAPs, particularly *Sac* aRNAP, involves coordinated multi-domain rearrangements.

The functional implications of these structural features align with the evolutionary divergence of transcription systems. Archaeal RNAP requires only two essential GTFs: TBP and TFB, supplemented by TFE, which stabilize cleft opening by bridging the clamp and stalk. In contrast, eukaryotic RNAP II must overcome both its intrinsic closed-cleft preference (Fig. 2) and a steric block created by the Rpb2 fork loop 1 insertion (Fig. 3), necessitating the coordinated action of multiple GTFs and coactivators. After the emergence of eukaryotes, RNAP function specialized into three paralogs: RNAP I and III each transcribe a limited number of genes with narrow functional roles, while RNAP II became responsible for producing the vast and highly regulated gene repertoire of eukaryotes (43). This expansion in regulatory complexity likely drove Pol II toward a more transcriptionally inactive basal state, increasing its dependence on GTFs to productively engage promoters. In contrast, archaeal RNAP - the ancestral enzyme from which all eukaryotic RNAPs evolved - must transcribe all gene classes using a single enzyme. This requirement may have preserved or even enhanced its intrinsic flexibility and dynamic range, enabling it to meet all cellular transcriptional demands without the elaborate GTF networks. These insights illuminate the evolutionary forces that shaped transcription initiation strategies across the tree of life and provide a foundation for future mechanistic and reconstitution studies aimed at understanding RNAP regulation in diverse systems.

## Experimental procedures

### *Pfu* aRNAP purification

*Pfu* aRNAP was purified as described (11).

### *S. acidocaldarius* strain and growth

Seed culture of the *S. acidocaldarius* strain expressing 6× His tagged at the C-terminus of Rpo8 (44) was prepared in a 1L Brock solution containing 20 μg/ml uracil, and 0.1% tryptone, and pH adjusted to 3 with 50% H_2_SO_4_. A 100 L fermentation was set up at the Penn State Huck Institutes of Life Science CSL-Behring Fermentation Facility in a nutrient-rich medium containing 0.3% tryptone, 0.6% sucrose, 0.1% yeast extract, and 20 μg/ml uracil. The cells were grown at 75 °C and pH 3.2 for ∼ 22 h and harvested at mid-exponential phase.

### *Sac* aRNAP purification

Cell paste was resuspended in lysis buffer (50 mM Tris-HCl pH 8.0, 100 mM NaCl, 1 mM DTT, 5% glycerol, and protein inhibitor cocktail) and lysed using a French press. Insoluble materials were removed by centrifugation at 18,000 RPM for 20 min, and the supernatant was applied to a HisTrap HP (Cytiva) equilibrated with Buffer A (10 mM Tris-HCl pH 8.0, 100 mM NaCl, 1 mM DTT, 5% glycerol) containing 25 mM imidazole. Proteins were eluted from Ni-column by Buffer A containing 250 mM imidazole. The eluted proteins were further purified by two successive column chromatographies including HiTrap Heparin, and HiTrap Q (Cytiva). Pooled fractions with *Sac* aRNAP were concentrated to ∼5 mg/ml.

### *S. cerevisiae* strain and growth

*S. cerevisiae* strain expressing Rpb3-GFP was generated from *S. cerevisiae* yM8.14 (45). GFP conjugation in yeast was carried out by amplifying GFP-KanMX from the pFA6a-GFP-KanMX plasmid and transformed using standard Lithium Acetate based transformation protocol to generate the final strain (*MATa his3-11 his3-15 leu2-3 leu2-112 can1 ura3-D5 pho80::HIS3 URA3::LEU2 pCYC1 LEXA-TAP pGAL1 RecR PHO5::RS_gene-3xLEXA RPB3-GFP::kanMX*). Final strains with Rpb3-GFP were verified by PCR genotyping and fluorescence imaging.

### Purification of *S. cerevisiae* eRNAP II

Six liters of YPAD yeast culture were grown to an OD_600_ 0.5 to 0.6 then harvested at 5000*g*’s for 10 min at 4 °C and resuspended in cold MiliQ water twice, before being resuspended in 50 to 100 ml of lysis buffer (20 mM HEPES-KOH pH 7.6, 200 mM KOAc, 10% Glycerol, 5 mM Mg(OAc)_2_, 0.5% Triton X-100, 0.1% Tween-20, 1 mM DTT, 1 mM PMSF, 50 mM Leupeptin, 2 μM Pepstatin A, 2 mM Benzamidine). The resuspension was spun down at 2700*g*’s for 15 min and the supernatant was removed twice to dry the pellet thoroughly. Crushed yeast noodles were then generated by pushing the dry pellet through a syringe into liquid nitrogen in a motor and broken up by a pestle. The resulting crushed noodles were processed through cryogenic grinding with a Retsch Cryomill (Retsch). To do so, the sample was cooled at five revolutions per second for 30 s and ground at 25 revolutions per second for 2 min. This last step was repeated four times. The resulting powder was stored in the −80 °C until further use.

The cell powder was resuspended in lysis buffer (20 mM Tris HCL pH 7.5, 200 mM KOAc, 5 mM Mg(OAc)_2_, 1 mM DTT, 1 mM PMSF) and spun down at 13,000*g* for 1 h at 4 °C. The resulting supernatant was saved and incubated with Dyna-pAb against GFP (Cristea *et al.*, 2005) overnight at 4 °C. The Dynabeads were pelleted by application of the mixture to a magnet. The pellet was washed by resuspension with cleavage buffer (same as lysis buffer without protease inhibitors) and end-over-end rotation for 10 min. This was repeated four times before resuspension in minimal volume and the addition of PreScission3C protease [0.1ug/uL final] for overnight cleavage at 4 °C. Sepharose 4B Glutathione (Cytiva) equilibrated with cleavage buffer was added to the elution and rotated end-over-end for 2 h at 4 °C. The mixture was passed through a mini chromatography column (Bio-Rad) to remove PreScission3C protease.

### Cryo-EM grid preparation and data acquisition

Quantifoil grids (R 2/1 Cu 300 Holey Carbon) were glow-discharged for 40 s followed by the application of a 3.5 μl sample containing RNAP (∼5 mg/ml) and 8 mM CHAPSO. The grids were plunge-frozen in liquid ethane using the FEI Mark IV Vitrobot set to 100% chamber humidity at 10 °C. Cryo-EM data were collected using an FEI Talos Arctica G2 operating at 200 kV with a Falcon 4 direct electron detector and Aberration-Free Image Shift at the Cryo-EM Facility at the Penn State Huck Institutes of the Life Sciences. Fifty frames per movie were collected for each sample, at a magnification of 150 k, corresponding to a pixel size of 0.94 Å, using 50 e^-^/A^2^ total dose and exposure time of 4.81 s.

### Cryo-EM data processing

All cryo-EM data were processed by CryoSPARC v4.1.2 (29). For each dataset, imported movies were patch motion-corrected, followed by manual exposure curation with adjusted threshold parameters. To generate a substate of particles for automatic particle picking, 100 micrographs were used for automatic blob picking with a particle diameter range of 100 Å to 200 Å. Only about a third of the particles were accepted owing to the stringent NCC threshold score. After particle extraction, several rounds of 2D classifications to eliminate junk particles. The selected particles and initially curated exposures for all movies were subjected to Topaz training (30) with 400 iterations to fit the model. A Topaz extraction job was then performed using the output from the initial training run to pick particles from the entire set of micrographs. The extraction process was carried out for 400 iterations to ensure sufficient time for model fitting. Particles were extracted and proceeded to multiple rounds of 2D classifications. Particles belonging to the best 2D classifications were selected and used to reconstruct reference-free *ab initio* 3D volumes, followed by 3D heterigrinous refinement to generate two maps corresponding to RNAP with and without the stalk domain. Final maps for *Pfu* aRNAP, *Sac* aRNAP, and yeast eRNAP II containing the stalk domain were obtained after a non-uniform refinement (Figs. S2–S4 and Tables S1–S4).

### Conformational analysis of RNAP

Subsequent 3D classification with a focused mask around the clamp domain (N = 10, filter resolution: 8 Å, per-particle scale: optimal), was performed for each RNAP dataset, which revealed two distinct classes, including the closed and open states of the clamp. The refined volumes of these classes were then used for heterogeneous refinement, where particles were subjected to force hard classification, with the batch size per class increased to 2000 to improve classification accuracy. The two subsets of ensembles generated from the heterogeneous refinement were reconstructed and subjected to non-uniform refinement (Figs S2–S4 and Tables S1–S4).

### Model building and refinement

Model building and refinements were carried out using Coot v0.9.8.95 (46) and Phenix v1.21.2 (47). Initial models for *Pfu* aRNAP and *Sac* aRNAP were built using AlphaFold (48), whereas a published model (PDB: 7MKA) (49) was used as a starting model for yeast eRNAP II. All models were manually fitted and refined in Coot against cryo-EM maps obtained from CryoSPARC. Further refinement was performed in Phenix with secondary structure restraint and validated using MolProbity (50). Cryo-EM map display and figure preparation were performed using USCF ChimeraX.

## Use of large language models

ChatGPT was used for grammatical correction of the text.

## Data and materials availability

All data needed to evaluate the conclusions of this paper are present in the Main Text and/or the Supplementary Materials. The cryo-EM maps and the refined models were deposited in the Electron Microscopy DataBank (EMDB, www.ebi.ac.uk/emdb/) and Protein Data Bank (PDB, www.rcsb.org): Stalk-bound: *P. furiosus* aRNAP closed clamp conformation (EMD-74465/PDB: 9ZO4) and open clamp conformation (EMD-74466/PDB: 9ZO5), *S. acidocaldarius* aRNAP closed clamp conformation (EMD-74490/PDB: 9ZOF) and open clamp conformation (EMD-74496/PDB: 9ZOH), *S. cerevisiae* eRNAP II closed clamp conformation (EMD-74528/PDB: 9ZPK) and open clamp conformation (EMD-74529/PDB: 9ZPL). Stalk-less: *P. furiosus* aRNAP (EMD-75066/PDB: 10CL), *S. acidocaldarius* aRNAP (EMD-75068/PDB: 10CN), *S. cerevisiae* eRNAP II (EMD-75178/PDB: 10HV).

## Supporting information

This article contains supporting information (9, 19, 33, 51).

## Conflict of interest

The authors declare that they have no conflicts of interest with the contents of this article.
