# Supplementary material for: Clamp conformational flexibility and dynamics in archaeal and eukaryotic RNA polymerases revealed by cryo-EM
Source: J Biol Chem. 2026 Aug 4;302(9):113389. doi: 10.1016/j.jbc.2026.113389 (PMC13545438; doi:10.1016/j.jbc.2026.113389)
Supplement: Supplementary file 1 [file mmc1.docx]

**Supporting Information**

**Title:** Clamp conformational flexibility and dynamics in archaeal and eukaryotic RNA polymerases revealed by cryo-EM

George N.R. Fordjour^1,2,3,4,5^, Leon Palao III^6^, Kenji Murakami^6^, Jean-Paul Armache^1,2,4^, Katsuhiko S. Murakami^1,2,3^

^1^Department of Biochemistry and Molecular Biology, Penn State University, University Park, PA 16802, USA

^2^Huck Institutes of the Life Sciences, Center for Structural Biology, Penn State University, University Park, PA 16802, USA

^3^Huck Institutes of the Life Sciences, Center for RNA Molecular Biology, Penn State University, University Park, PA 16802, USA

^4^Huck Institutes of the Life Sciences Center for Eukaryotic Gene Regulation, Penn State University, University Park, PA 16802, USA

^5^Molecular Machines Mechanism and Structure Predoctoral Training Program, Penn State University, University Park, PA 16802, USA

^6^Department of Biochemistry and Biophysics, Perelman School of Medicine, University of Pennsylvania, Philadelphia, PA 19104, USA

Keywords: cellular RNA polymerase, cryo-electron microscopy, conformational dynamics, promoter complex

*Corresponding authors Email: [kum14@psu.edu](mailto:kum14@psu.edu), [jza449@psu.edu](mailto:jza449@psu.edu)

**Supplementary Fig. 1: Structures and subunit compositions of archaeal and eukaryotic RNAPs.**

Cryo-EM maps of the archaeal RNAPs (*Pyrococcus furiosus, Pfu*, left; *Sulfolobus acidocaldarius, Sac*, middle) and eukaryotic RNAP II (*Saccharomyces cerevisiae*, yeast, right). Each subunit is depicted by a unique color, and orthologous subunits are depicted with the same color. Functions of these subunits are listed at the bottom panel.

**Supplementary Fig. 2: Cryo-EM workflow for the *Pfu* aRNAP data processing.**

(**A**) A representative micrograph. (**B**) Selected 2D classes. (**C**) Cryo-EM data processing and classification flow chart. Pink mesh depicts the mask around the RNAP clamp during 3D classification.

**Supplementary Fig. 3: Cryo-EM workflow for the *Sac* aRNAP data processing.**

(**A**) A representative micrograph. (**B**) Selected 2D classes. (**C**) Cryo-EM data processing and classification flow chart.

**Supplementary Fig. 4: Cryo-EM workflow for the yeast eRNAP II data processing**

(**A**) A representative micrograph. (**B**) Selected 2D classes. (**C**) Cryo-EM data processing and classification flow chart.

**Supplementary Fig. 5: Attempts to separate Sac aRNAP lacking the stalk domain.**
(A) Purification of *Sac* aRNAP by Ni–NTA affinity chromatography followed by size-exclusion chromatography (SEC). Elution profile is shown, and peak fractions were collected for further analysis. (B) Peak fraction (fraction 2) from the initial SEC was subjected to a second round of SEC to improve separation. (C, D) SDS–PAGE analysis of fractions from the first (C) and second (D) chromatography steps. Individual RNAP subunits are indicated. (E, F) Quantification of band intensities from SDS–PAGE gels in (C) and (D), respectively, using ImageJ (4). Relative abundances of stalk subunits Rpo7 (blue) and Rpo4 (green) were measured using Rpo3 (gray) and Rpo8 (yellow) as references, respectively. Despite reduced signal intensity of the stalk subunits (Rpo7 and Rpo4) relative to core RNAP components in some fractions, these subunits were consistently detected across all fractions, indicating that complete separation of stalk-deficient aRNAP was not achieved under the tested conditions.

**Supplementary Fig. 6. Quantification of clamp opening and rotation.**

(**A**) Measurement of DNA-binding cleft width using the minimum distance between the fork loop and rudder in the closed (green) and open (red) clamp states (representative positions indicated). (**B**) Measurement of clamp rotation as the angular displacement of the distal tip of the clamp helix relative to the hinge (“switch”) region, comparing closed (green) and open (red) states. (**C**) Residues used as reference points for quantifying clamp rotation and cleft widening in *Pfu* aRNAP, *Sac* aRNAP, and yeast eRNAP II. Cα was used for all measurements.

**Supplementary Fig. 7. Structural changes associated with clamp opening in archaeal and eukaryotic RNAPs.**

Closed- and open-clamp conformations of *Pfu* aRNAP (left), *Sac* aRNAP (middle), and yeast eRNAP II (right) are superimposed. Structural differences between the two states are mapped onto the models as per-residue RMSD (Å), highlighting regions that undergo notable rearrangements during clamp opening (color scale).

**Supplemental Fig. 8: Illustration of the mobile modules of the RNAPs.**

(**A-C**) Structural modules of RNAPs are shown with orientations adjusted relative to **Fig. 4a** to emphasize conformational mobility. (**A**) Stalk module rotated 330°. (**B**) Jaw module rotated 30°**.** (**C**) Lobe module rotated 330°.

**Supplementary Table S1**: Cryo-EM Data collection, refinement, and validation statistics *Pfu* aRNAP dataset

| Data collection | | | |
| --- | --- | --- | --- |
| Magnification | 150,000 | | |
| Voltage (kV) | 200 | | |
| Electron dose (e^-^/Å^2^) | 50 | | |
| Number of micrographs | 5,000 | | |
| Image pixel size (Å) | 0.944 | | |
| Defocus range (μm) | -0.8, -2.2 | | |
| Symmetry imposed | C1 | | |
| Final particle (no.) | Stalk-bound form  293,245 | | Stalk-less form (no Rpo4/7)  61,865 |
| RNAP conformation | Closed  150,366 | Open  142,876 |  |
| PDB | 9ZO4 | 9ZO5 | 36RO |
| EMBD | EMD-74465 | EMD-74466 | EMD-77809 |
| Refinement | | | |
| Initial model used (PDB code) | AlphaFold | AlphaFold | AlphaFold |
| Map resolution (Å)  FSC threshold 0.143 | 4.05 | 4.05 | 4.56 |
| Model composition |  |  |  |
| Non-Hydrogen Atoms | 26344 | 26194 | 23712 |
| Protein residues | 3287 | 3266 | 2961 |
| Ligands | Zn:5 | Zn:5 | Zn:5 |
| Bonds (RMSD) |  |  |  |
| Length (Å) (# > 4σ) | 0.004 | 0.003 | 0.003 |
| Angles (°) (# > 4σ) | 0.662 | 0.706 | 0.741 |
| ADP (B-factors) |  |  |  |
| Protein | 107.78 | 86.11 | 340.51 |
| Ligand | 179.50 | 171.61 | 469.91 |
| Validation |  |  |  |
| MolProbity score | 1.86 | 1.84 | 1.88 |
| Clash score | 9.64 | 8.95 | 10.25 |
| Rotamer outliers (%) | 0.00 | 0.00 | 0.00 |
| Ramachandran plot |  |  |  |
| Favored (%) | 94.97 | 94.75 | 94.93 |
| Allowed (%) | 4.90 | 5.06 | 4.93 |
| Outliers (%) | 0.12 | 0.19 | 0.14 |

**Supplementary Table 2:** Cryo-EM Data collection, refinement, and validation statistics of *Sac* aRNAP dataset

| Data collection | | | |
| --- | --- | --- | --- |
| Magnification | 150,000 | | |
| Voltage (kV) | 200 | | |
| Electron dose (e^-^/Å^2^) | 50 | | |
| Number of micrographs | 3,079 | | |
| Image pixel size (Å) | 0.944 | | |
| Defocus range (μm) | -1.0, -2.4 | | |
| Symmetry imposed | C1 | | |
| Final particle (no.) | Stalk-bound form  275,155 | | Stalk-less form (no Rpo4/7)  27,665 |
| RNAP conformation | Closed  194,130 | Open  81,025 |  |
| PDB | 9ZOF | 9ZOH | 36RP |
| EMBD | EMD-74490 | EMD-74496 | EMD-77810 |
| Refinement | | | |
| Initial model used (PDB code) | AlphaFold | AlphaFold | AlphaFold |
| Map resolution (Å)  FSC threshold 0.143 | 3.37 | 3.87 | 8.9 |
| Model composition |  |  |  |
| Non-Hydrogen Atoms | 27395 | 27273 | 25012 |
| Protein residues | 3457 | 3441 | 3154 |
| Ligands | SF4:1; Zn:5 | SF4:1; Zn:5 | SF4:1; Zn:5 |
| Bonds (RMSD) |  |  |  |
| Length (Å) (# > 4σ) | 0.003 | 0.005 | 0.006 |
| Angles (°) (# > 4σ) | 0.559 | 0.666 | 1.199 |
| ADP (B-factors) |  |  |  |
| Protein | 125.74 | 118.68 | 512.51 |
| Ligand | 121.86 | 127.80 | 518.85 |
| Validation |  |  |  |
| MolProbity score | 1.53 | 1.83 | 2.04 |
| Clash score | 4.33 | 6.62 | 11.14 |
| Rotamer outliers (%) | 0.00 | 0.00 | 0.00 |
| Ramachandran plot |  |  |  |
| Favored (%) | 95.45 | 92.64 | 92.29 |
| Allowed (%) | 4.52 | 7.33 | 7.65 |
| Outliers (%) | 0.03 | 0.03 | 0.06 |

**Supplementary Table 3:** Cryo-EM Data collection, refinement, and validation statistics of yeast, *Saccharomyces cerevisiae* eRNAP II dataset

| Data collection | | | |
| --- | --- | --- | --- |
| Magnification | 150,000 | | |
| Voltage (kV) | 200 | | |
| Electron dose (e^-^/Å^2^) | 50 | | |
| Image pixel size (Å) | 0.944 | | |
| Defocus range (μm) | -0.8, -2.2 | | |
| Symmetry imposed | C1 | | |
| Final particle (no.) | Complete form  267,768 | | No stalk (no Rpo4/7)  39,868 |
| RNAP conformation | Closed  208,045 | Open  59,723 |  |
| PDB | 9ZPK | 9ZPL | 36RS |
| EMBD | EMD-74528 | EMD-74529 | EMD-77811 |
| Refinement | | | |
| Initial model used (PDB code) | 7MKA | 7MKA | 7MKA |
| Map resolution (Å)  FSC threshold 0.143 | 3.31 | 3.75 | 6.75 |
| Model composition |  |  |  |
| Non-Hydrogen Atoms | 31439 | 30373 | 28625 |
| Protein residues | 3965 | 3826 | 3608 |
| Ligands | Zn:8 | Zn:7 | Zn:8 |
| Bonds (RMSD) |  |  |  |
| Length (Å) (# > 4σ) | 0.005 | 0.003 | 0.005 |
| Angles (°) (# > 4σ) | 0.927 | 0.710 | 0.980 |
| ADP (B-factors) |  |  |  |
| Protein | 101.04 | 221.81 | 369.15 |
| Ligand | 144.95 | 323.40 | 610.77 |
| Validation |  |  |  |
| MolProbity score | 1.94 | 2.01 | 2.19 |
| Clash score | 7.60 | 8.79 | 13.22 |
| Rotamer outliers (%) | 0.00 | 0.00 | 0.00 |
| Ramachandran plot |  |  |  |
| Favored (%) | 90.80 | 90.35 | 89.69 |
| Allowed (%) | 9.02 | 9.28 | 9.970 |
| Outliers (%) | 0.18 | 0.37 | 0.34 |

**Supplementary Table 4:** Distance of the DNA-binding cleft archaeal RNAPs determined by this study and previous studies.

|  | PDB ID | Distance (Å) | reference |
| --- | --- | --- | --- |
| *Pfu* aRNAP |  |  | This study |
| closed | 9ZO4 | 14.8 |  |
| open | 9ZO5 | 29.6 |  |
|  |  |  |  |
| *Sac* aRNAP |  |  | This study |
| closed | 9ZOF | 16.3 |  |
| open | 9ZOH | 22.1 |  |
|  |  |  |  |
| Yeast eRNAP II |  |  | This study |
| closed | 9ZPK | 16.3 |  |
| open | 9ZPL | 30.9 |  |
|  |  |  |  |
| *Tko* aRNAP |  |  | (1) |
| apo-form | 6KF3 | 14.8 |  |
| with TFE | 6KF4 | 25.1 |  |
| with TEF+DNA | 6KF9 | 13.0 |  |
|  |  |  |  |
| *Pfu* aRNAP |  |  | (2) |
| contracted form | 8RBO | 14.6 |  |
| expanded form | 8ORQ | 31.4 |  |
| EC | 8CRO | 14.8 |  |
|  |  |  |  |
| *Sac* aRNAP |  |  | (3) |
| apo-form | 7OK0 | 18.0 |  |
| with TFS4 | 7OQY | 20.1 |  |

The distance of DNA-binding cleft of *Thermococcus kodakarensis*, *Tko* RNAP was measured using Rpo1/Gly298 and Rpo2/Gly411.

**REFERENCES**

1.	Jun, S.-H., Hyun, J., Cha, J. S., Kim, H., Bartlett, M. S., Cho, H.-S. *et al.* (2020) Direct binding of TFEα opens DNA binding cleft of RNA polymerase Nature Communications 2020 11:1 **11**, 10.1038/s41467-020-19998-x

2.	Tarau, D., Grunberger, F., Pilsl, M., Reichelt, R., Heiss, F., Konig, S. *et al.* (2024) Structural basis of archaeal RNA polymerase transcription elongation and Spt4/5 recruitment Nucleic Acids Res **52**, 6017-6035 10.1093/nar/gkae282

3.	Pilotto, S., Fouqueau, T., Lukoyanova, N., Sheppard, C., Lucas-Staat, S., Díaz-Santín, L. M. *et al.* (2021) Structural basis of RNA polymerase inhibition by viral and host factors Nature Communications 2021 12:1 **12**, 10.1038/s41467-021-25666-5

4.	Schneider, C., Rasband, W. & Eliceiri, K. NIH Image to ImageJ: 25 years of image analysis. Nat Methods 2012 **9**, 671–675 10.1038/nmeth.2089
